# Single-Cell Visualization Deep in Brain Structures by Gene Transfer

**DOI:** 10.3389/fncir.2020.586043

**Published:** 2020-11-19

**Authors:** Sayaka Sugiyama, Junko Sugi, Tomoya Iijima, Xubin Hou

**Affiliations:** Laboratory of Neuronal Development, Graduate School of Medical and Dental Sciences, Niigata University, Niigata, Japan

**Keywords:** single-cell electroporation, overexpression, visual cortex, thalamus, lateral pulvinar, presynaptic protein, postnatal development

## Abstract

A projection neuron targets multiple regions beyond the functional brain area. In order to map neuronal connectivity in a massive neural network, a means for visualizing the entire morphology of a single neuron is needed. Progress has facilitated single-neuron analysis in the cerebral cortex, but individual neurons in deep brain structures remain difficult to visualize. To this end, we developed an *in vivo* single-cell electroporation method for juvenile and adult brains that can be performed under a standard stereomicroscope. This technique involves rapid gene transfection and allows the visualization of dendritic and axonal morphologies of individual neurons located deep in brain structures. The transfection efficiency was enhanced by directly injecting the expression vector encoding green fluorescent protein instead of monitoring cell attachment to the electrode tip. We obtained similar transfection efficiencies in both young adult (≥P40) and juvenile mice (P21–30). By tracing the axons of thalamocortical neurons, we identified a specific subtype of neuron distinguished by its projection pattern. Additionally, transfected mOrange-tagged vesicle-associated membrane protein 2–a presynaptic protein—was strongly localized in terminal boutons of thalamocortical neurons. Thus, our *in vivo* single-cell gene transfer system offers rapid single-neuron analysis deep in brain. Our approach combines observation of neuronal morphology with functional analysis of genes of interest, which can be useful for monitoring changes in neuronal activity corresponding to specific behaviors in living animals.

## Introduction

Brain networks are shaped by experience during postnatal development. Neuronal morphology–including dendrites and axon–is highly diverse and closely related to functional connectivity in neural circuits. Even neurons that are located close to each other in a given brain area differ in their axonal projection patterns and participate in distinct circuits (Jones, 2001; Clascá et al., 2012). As such, visualization of neuronal morphology as well as connectivity–which is the basis of brain function–has been a focus of neuroscience research since the development of the Golgi staining method.

Recent progress in imaging technologies and big data analysis has enabled brain-wide mapping and reconstruction of fluorescence-labeled neurons. Connectome analyses have revealed extensive projections in many brain areas (Livet et al., 2007; Cai et al., 2013), contacts among neurons within a sensory pathway (Bock et al., 2011; Morgan et al., 2016), and different cortical neuron subtypes that share physiologic and genetics features (Gouwens et al., 2019). Thus, advances in data science have contributed to the elucidation of complex neural circuitry (Lichtman et al., 2014; Winnubst et al., 2019).

Brain mapping depends on the ability to visualize small populations of neurons. Retrograde tracing of monosynaptic circuits has been carried out using a deletion mutant rabies virus to label a subset of neurons through Cre-dependent marker expression (Wickersham et al., 2007). Single-cell electroporation of recombinant rabies virus combined with 2-photon imaging enabled transsynaptic tracing of a single neuron in the cerebral cortex (Kitamura et al., 2008; Marshel et al., 2010).

The electroporation technique, which was originally developed in embryos (Muramatsu et al., 1997; Funahashi et al., 1999; Nakamura, 2009), has been employed in innovative ways in neuroscience research. In mitotic cells of the embryonic neuroepithelium, expression of an exogenous gene introduced via electroporation can be detected 1 day later, and rapidly expands as cells proliferate. In contrast, in the postnatal brain the efficiency of gene transfer–especially in postmitotic neurons–is reduced, requiring modification of the standard protocol of juxtacellular labeling (Oyama et al., 2013; Ohmura et al., 2015). Electroporation has been adopted in gain- and loss-of-function studies [e.g., using short hairpin (sh)RNAs or the clustered regularly interspaced short palindromic repeats (CRISPR)/CRISPR-associated protein (Cas)9 system] and to observe neuronal morphology in the complex brain of gyrencephalic mammals (Matsui et al., 2013; Shinmyo et al., 2017). The long-lasting expression of the calcium sensor protein GCaMP or potassium channel Kir2.1 permits the examination of neuronal responses to a certain stimulus (Pagès et al., 2015; Mizuno et al., 2018) or of activity-dependent effects on neuronal morphology and function (Mizuno et al., 2007; De Marco García et al., 2011; Hagihara et al., 2015). Importantly, the response of individual neurons within a circuit to a stimulus varies according to the neuronal subtype. Despite the availability of single-neuron analysis in the cerebral cortex (Kitamura et al., 2008; Judkewitz et al., 2009; Pagès et al., 2015), individual neurons deep in brain structures remain difficulty to analyze. Thus, novel approaches for examining neural circuitry with single-cell resolution, especially within deep brain structures, are needed.

To this end, in the present study we developed an efficient technique for single-cell electroporation in the juvenile and adult mouse brain. The method enabled rapid postnatal transfection of single neurons deep within the brain and visualization of neuronal morphology, including thalamocortical axons. Electroporation of an mOrange-tagged vesicle-associated membrane protein (VAMP)2 vector revealed localization of this presynaptic protein in terminal boutons of thalamocortical axons. This technique in combination with high-resolution imaging systems expands the ability to analyze neural circuits derived from deep brain structures with single-cell resolution.

## Materials and Methods

### Animals

Animal experiments were performed in accordance with the protocol approved by the Committee for Animal Care at Niigata University (reference no. SA00534). C57Bl/6J mice of both sexes were purchased from Japan SLC (Hamamatsu, Japan) and reared under standard conditions. Mice of the same sex were housed in groups of 2–3 per cage (143 mm × 293 mm × 148 mm, Charles River) and maintained on a 12:12-h light/dark cycle with free access to food and water. Mice were used for electroporation at 3–8 weeks old.

### *In vivo* Single-Cell Electroporation

**FIGURE 1:**
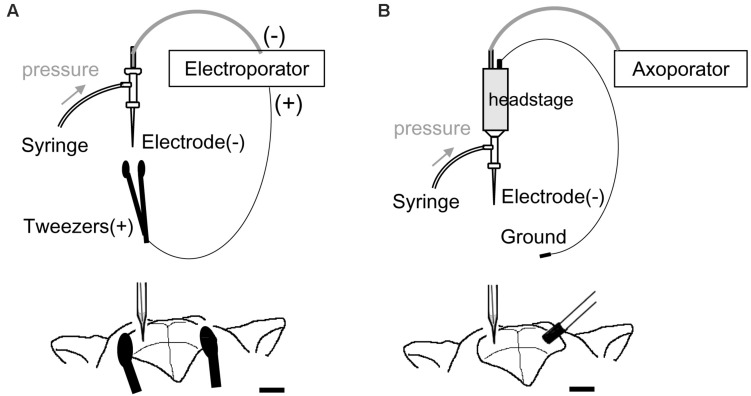
Schematic illustration of electroporation and electrodes system. **(A)** Electroporation setup modified from the embryonic system. A sharp glass electrode containing the DNA solution was connected to the negative terminal of the electroporator and inserted into the target region. Tweezer-type electrode was connected into the positive terminal of the electroporator and placed outside the skull. **(B)** Electroporation setup modified from single-cell electroporation. A sharp glass capillary (negative electrode) was inserted into the target region and a ground electrode was placed over the contralateral hole in the skull. The glass capillary and ground electrode were connected to the headstage.

VAMP2 cDNA was obtained from Clontech/Takara Bio (Otsu, Japan). pCAGGS-enhanced green fluorescent protein (EGFP) (Hou et al., 2013) and pT2K-CAGGS-VAMP2-mOrange (Sato et al., 2007; Egawa et al., 2013) vectors were purified using a plasmid purification kit (Qiagen, Hilden, Germany) and dissolved in Tris–EDTA buffer (1 μg/μl). A glass capillary tube (internal diameter, 0.75 mm; external diameter, 1 mm; B100-75-10PT; Sutter Instrument, Novato, CA, United States) was pulled using a micropipette puller (taper length, 7–8 mm with P-1000 micropipette puller program, Sutter Instrument). We cut the tip of the capillary using scissors so that the external diameter was 2–50 μm. The capillary was used for 1 to 3 penetrations before it got clogged. The glass capillary was filled with 1 μl DNA solution and inserted into a micropipette holder, with a silver chloride wire connected via a pin (Molecular Devices, San Jose, CA, United States) serving as the cathode (Figure 1). We applied positive air pressure (100–300 mbar for weak pressure, 300–1,000 mbar for strong pressure) using a 1-ml syringe connected to the micropipette holder to inject DNA solution (estimated <0.1 μl by weak pressure, 0.3–0.5 μl by strong pressure). For electroporation, mice were anesthetized under isoflurane. Standard stereotactic procedures were used for surgery. The glass electrode containing DNA solution was connected to the micromanipulator and advanced into the brain through a 1–2 mm diameter hole, which was made on each side of the skull [2.5 mm lateral and 2 mm caudal to bregma for the lateral geniculate nucleus (LGN)/lateral posterior nucleus (LP)/hippocampus, 0.5 mm lateral and 2.5 mm caudal to bregma for the ventral tegmental area (VTA), and 2.5 mm lateral and 3.5 mm caudal to bregma for the primary visual cortex (V1)]. For our modified electroporation method from embryonic system (Figure 1A), we used tools for embryonic electroporation, a 3-mm diameter tweezer-type electrode (BEX, Tokyo, Japan) as the anode and an electroporator (CUY21EDIT, Bex). In this system, negatively charged DNA moves toward the anode in the electric field and can enter the cell around the tip of the glass electrode. To this end, the plates of the tweezer-type electrode were placed outside on either side of the skull and a square pulse (100–150 V for 1 ms at 200 Hz, for a total duration of 500 ms) was delivered 3–5 times at different depths. The resistance and current flow between the cathode and anode (monitored during electrical stimulation with the CUY21 electroporator) were ∼0.018 MΩ and 10 mA, respectively. The current was lower than the 40–60 mA that are usually applied for *in utero* electroporation. In a modification from the single-cell electroporation methods (Figure 1B), a 1-mm diameter ground electrode (Ag/AgCl pellet; Molecular Devices) connected to AP-1A headstage was used. Immediately after DNA injection, the ground electrode was inserted into the contralateral hole and a square pulse (80 V for 1 ms at 200 Hz, for a total duration of 3 s) was delivered 3–5 times at different depths (Axoporator 800A, Molecular Devices). For electroporation into cortical neurons, mannitol was intraperitoneally injected (250 μl, 200 mg/ml) to decrease the pressure in the brain. After the electroporation procedure, mice were returned to their home cage until the next experiment.

### Immunohistochemistry

Immunolabeling was performed as previously described (Sugiyama et al., 2008). Serial coronal sections (50 μm) were cut and incubated with anti-GFP (mouse monoclonal IgG2a, mFX73; Wako, Osaka, Japan) and anti-tyrosine hydroxylase (TH; mouse monoclonal IgG1α, LNC1; Millipore, Billerica, MA, United States) antibodies. Alexa Fluor 488 or 594-conjugated anti-mouse IgG (Invitrogen, Carlsbad, CA, United States) was used as secondary antibodies.

### Analysis of Neuronal Morphology and Projections

For observation of neuronal morphology, images were captured under a confocal microscopy (excitation wavelength, 488 and 543 nm) and multiple planes were combined to Z-projections (Nikon, Tokyo, Japan). For axon tracing, serial sections were mounted in sequence and images were automatically captured under a stereomicroscope (Keyence, Osaka, Japan) using a tiling protocol. Axonal fibers were reconstructed in two dimensions section by section; serial images were superimposed, and neuronal processes were traced using ImageJ software (National Institutes of Health, Bethesda, MD, United States). For quantitative analysis of VAMP2-mOrange localization, VAMP2-mOrange positive puncta were defined by combining the threshold (between the intensity values of 60 to 255) and the length (above 2 μm) to distinguish from the background signal. The number of VAMP2-mOrange puncta was counted per dendritic or axonal segment and compared between dendrites and axons (Mann–Whitney *U* test). All statistical values obtained from morphological experiments are presented as means ± standard error of the mean (SEM).

## Results

### Modified Single-Cell Electroporation in Postnatal Thalamus

**FIGURE 2:**
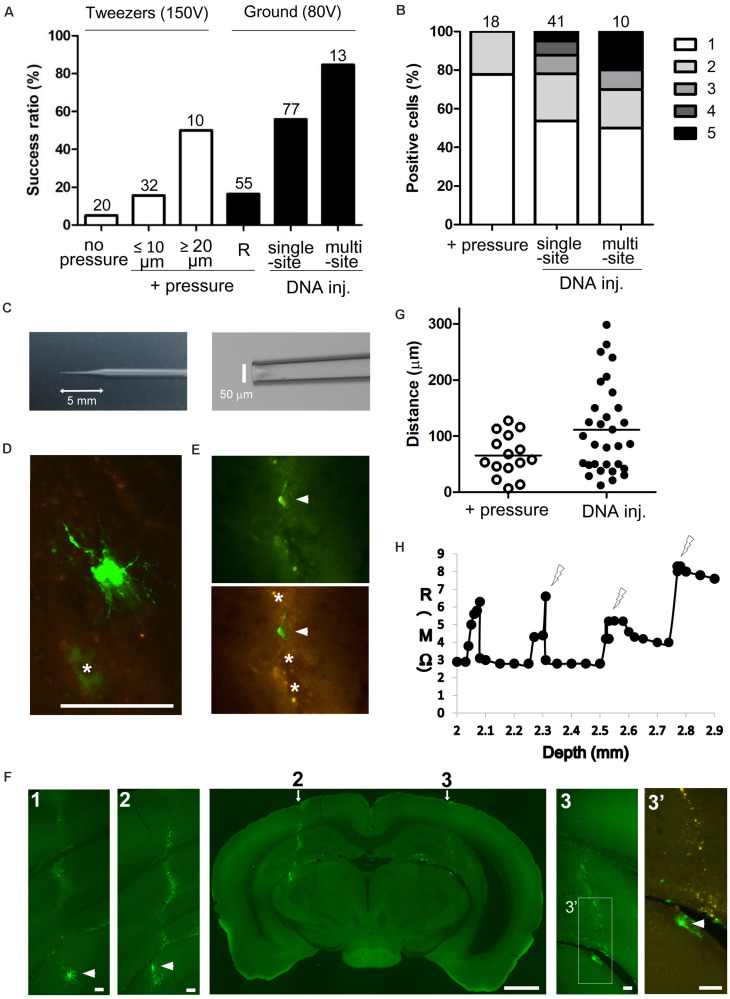
Gene delivery to deep brain neuron by single-cell electroporation. **(A)** Electroporation efficiency under different conditions. Percentage of GFP-expressing sites per penetration site (1 penetration site per target area) is shown; in the case of multiple-site injection, the percentage of GFP-expressing areas per target area (3 penetration tracts per target area) is shown. Total number of penetration sites (or total number of target areas for multiple-site injection) is given above each bar. + pressure, electroporation under weak pressure; DNA injection, electroporation after injecting DNA under strong pressure; R, electroporation by monitoring tip and cleft resistance. **(B)** The number of GFP-expressing cells per GFP-positive site is shown; in the case of multiple-site injection, the number of GFP-expressing cells per GFP-positive target region is shown. The box color (white to black) indicates the number of GFP-expressing cells. Total number of GFP-positive sites (or total number of GFP-positive regions for multiple-site injection) is given above each bar. **(C)** A sharp glass electrode with a 5-mm length of shank and 50-μm tip diameter prepared by cutting the glass tip. **(D,E)** GFP-expressing cells 2 days after electroporation. Most of transfected cells showed intense green fluorescence **(D)**, but we found damaged cells (arrowheads in panel **E**) with weak GFP and poor processes near the penetration sites (asterisks). The penetration sites exhibited green and red autofluorescence. **(F)** Whole brain slice including two penetration tracts (arrows) and GFP-expressing cells (arrowheads) at both sides. Autofluorescence was detected along the penetration tracts (**2** and **3**, higher magnification view of areas indicated by arrows; **1**, view of a serial section at a 100 μm distance from panel **2**, **3’**, higher magnification view of areas indicated in panel **3** with green and red fluorescence) **(G)** Distance of GFP-expressing cells from the penetration tracts. GFP-expressing cells were detected far from penetration sites after strong pressure DNA injection than after weak air pressure (“+ pressure”) (*p* = 0.0115, *t*-test with Welch’s correction; *p* < 0.05, χ^2^-test). **(H)** Relationship between tip and cleft resistance (R) and depth of the electrode from the pia mater. As resistance increased, square pulses were delivered at the points indicated by the lightning shape. Scale bars, 1 mm for panel **(F)**; 100 μm for panels **(D,E,F1–3’)**.

**FIGURE 3:**
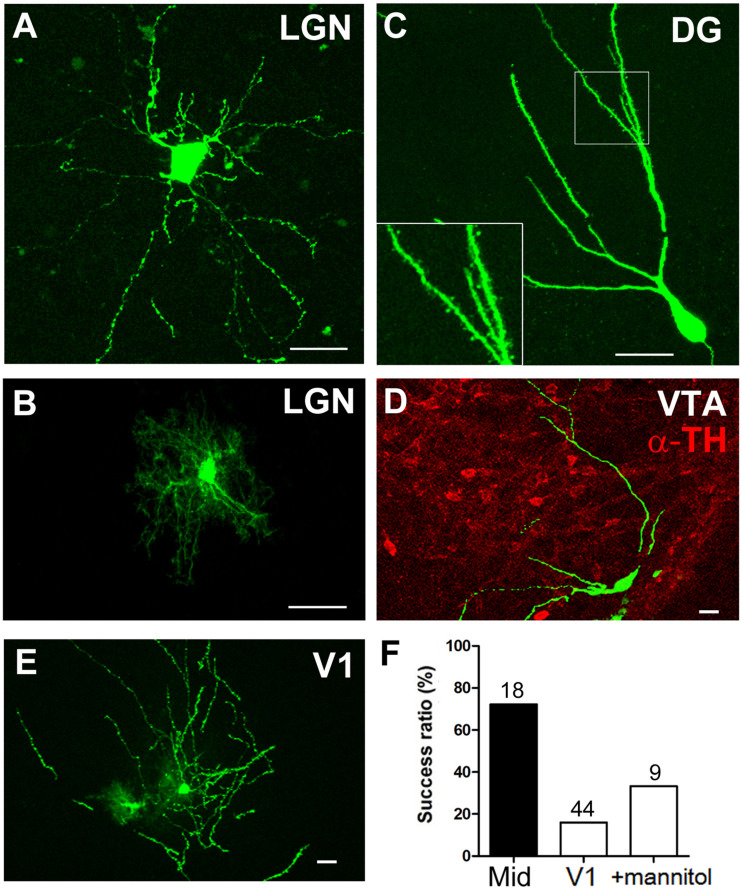
Dendrite morphology of single cells labeled by postnatal electroporation. **(A–E)** Cell body and dendrites of GFP-expressing cells in the LGN (**A**, neuron; **B**, glia), DG **(C)**, VTA **(D)**, and V1 **(E)**. Dendritic spines were clearly visible in the granule cell (**C**, inset). The VTA of the midbrain was identified based on TH immunoreactivity (**D**, red). Scale bars, 20 μm. **(F)** Transfection efficiency in the midbrain and V1. The percentage of GFP-expressing sites per penetration site after single-site DNA injection and electroporation is shown. Total number of penetration sites is given above each bar.

We first optimized the electroporation procedure to achieve maximum transfection efficiency by electroporating a GFP vector (pCAGGS-EGFP) into thalamic neurons of juvenile (P21–30) and adult (≥P40) mouse brains. In embryos, the efficiency of electroporation is mostly dependent on DNA concentration and electric current around target cells (Nakamura, 2009). We therefore tested different conditions for DNA injection and electrical stimulation (Figures 1, 2). As the sharp glass electrode (length of shank, 5–7 mm) was penetrated into the thalamic region, weak positive pressure was applied by pushing 0.1–0.2 cm^3^ air (100–300 mbar) through a 1-ml syringe connected to the electrode holder (“+ pressure” in Figure 2A), as in the standard patch-clamp method. Then, while holding both sides of the skull between the plates of the tweezer-type electrode, a square pulse (100–150 V for 1 ms at 200 Hz, for a total duration of 500 ms) was delivered five times at different depths (at 100-μm intervals in target area). The electroporation efficiency was increased by ejecting the DNA solution (estimated <0.1 μl) using positive pressure (1 positive site/20 penetration sites for “no pressure” vs. 19/97 positive sites for “+ pressure” under three different conditions in Figure 2A). In both cases, there were a few GFP-expressing cells around the injection site (Figure 3), and single labeled cells were observed in most cases (79% positive penetration sites for “+pressure” in Figure 2B). Importantly, the transfection efficiency was higher with electrodes with a tip diameter of 20–25 μm (i.e., ≥20 μm) as compared to 2–10 μm (i.e., ≤10 μm) (5/10 sites for diameter ≥20 μm vs. 5/32 sites for diameter ≤10 μm with pressure; Figure 2A).

In order to maximize the amount of DNA around targets cells, the tip of the glass capillary was cut to an external diameter of up to 50 μm (length of shank, 5–6 mm; Figure 2C), and DNA solution (0.3–0.5 μl) was injected under a strong air pressure (300–1,000 mbar, i.e., until the level of solution in the electrode was visually confirmed to decrease). Immediately after DNA injection the ground electrode (Ag/AgCl pellet, 1 mm diameter) was positioned in the contralateral hole of the skull (Figure 1B) and square pulses (80 V for 1 ms at 200 Hz, for a total duration of 3 s) were delivered 3–5 times at different penetration depths (at 100-μm intervals; for LGN, injecting DNA at a depth of 2.8 mm and delivering pulses at depths of 2.7, 2.8, and 2.9 mm). As expected, the transfection efficiency was higher with strong air pressure (43/77 penetration sites for single-site DNA injection; Figure 2A) than that with weak air pressure (“+pressure”) and was similar between young adult and juvenile mice (6/8 sites for ≥P40 mice, 37/69 sites for P21–P30 mice). The number of labeled cells were also increased at the positive sites (for single-site DNA injection in Figure 2B) than at sites of weak pressure (“+ pressure”), and we found single-labeled cells in 54% of positive sites. To further improve the efficiency of gene transfer, a set of a DNA injection and 3–5 electrical pulse deliveries per penetration site (“single-site DNA injection”) was repeated along three different penetration tracts (at 200-μm intervals in rostrocaudal or mediolateral axes) within the target area (“multisite DNA injection”). Multisite DNA injections achieved a GFP-expressing cell ratio per target area of 85% (11 positive areas/13 target areas for multisite DNA injection in Figure 2A). Single labeled cells were observed in 50% of positive areas (for multisite DNA injection in Figure 2B).

We found false positive cells or dead cells exhibiting strong autofluorescence along the penetration tracts of the glass electrode as is common while using other injection methods (“+ pressure” in Figures 2D,E, “DNA injection” in Figure 2F). Although cellular damage was observed along the two penetration paths toward the GFP-expressing LGN cells, neither DNA injection nor electrical pulse deliveries caused large lesions on a whole slice (Figure 2F; panels 1, and 2 after single-site injection and panel 3 after multisite injection). Damaged cells weakly expressing GFP and exhibiting poor processes were sometimes detected near the injection sites (Figure 2E). Maximizing the amount of DNA by applying strong air pressure did not increase the damaged-cell ratio (1/14 labeled cells for single-site DNA injection) when compared with the ratio after applying weak pressure (2/23 labeled cells for “+ pressure”) 1–6 days after electroporation. Moreover, the transfection efficiency (50%) after single-site DNA injection had not declined 3–4 weeks after electroporation (11/22 sites after 1–6 days vs. 31/61 sites after 3–4 weeks), suggesting that transfected cells were viable for at least a month. Consistent with the high transfection efficiency, DNA injection resulted in an increase in the number of GFP-expressing cells far from the injection sites (Figure 2G; + pressure, 65.48 ± 9.69 μm vs. DNA injection, 110.90 ± 14.21 μm, 15–31 cells, *p* = 0.0115, *t*-test with Welch’s correction; *p* < 0.05, χ^2^-test with 1, 5, 10, or 20 μm bin). Thus, cell damages were minimized by using the long-shank electrode for DNA injection and by delivering an appropriate electronic pulse (40–200 μA amplitude around the tip of the glass electrode, according to Uesaka et al., 2008).

We next evaluated whether cell attachment to the tip of the electrode could increase the efficiency of gene transfer during the single-cell electroporation (Haas et al., 2001; Kitamura et al., 2008; Oyama et al., 2013). To measure cleft resistance between the electrode tip and presumptive cell membrane, a glass electrode (2–5 μm tip diameter) and ground electrode were connected to the headstage of the electroporator (Axoporator 800A). As the tip of the electrode approached the cell membrane, tip and cleft resistance increased, mimicking seal formation; when this occurred, a square pulse (100 V for 1 ms at 200 Hz, for a total duration of 3 s) was delivered (Figure 2H). As a result, GFP-expressing cells were observed at 16% of penetration sites (9/55 sites for “R” in Figure 2A). As the other electroporation conditions were similar to those used when ejecting DNA under a weak air pressure (≤10 μm), the transfection efficiency was similar regardless of the cleft resistance (Figure 2A). Thus, DNA ejection from an electrode with a tip diameter greater than 20 μm improves the efficiency of gene transfection into postmitotic cells.

### Visualization of Cell Bodies and Dendrites in the Brain

To confirm the effectiveness of the electroporation system in different brain regions, a GFP-encoding plasmid was electroporated into V1, dentate gyrus (DG), and VTA as well as the thalamus (Figure 3). Confocal images revealed GFP expression in neurons and glia within the LGN of the thalamus; the cell body and neuronal processes were visible 1 day after electroporation (Figures 3A,B). Similarly, the dendritic spines of GFP-expressing granule cells of the DG were clearly observed (Figure 3C). Comparable single-cell labeling was achieved in the midbrain VTA (Figure 3D), and the transfection efficiency was slightly higher than in the thalamus after single-site DNA injection (Figure 3F, 13 positive sites/18 penetration sites). On the other hand, although there were a few GFP-expressing cells (Figure 3E), the transfection efficiency in the cortex was lower than in the other brain regions (7 positive sites/44 penetration sites). As DNA solution easily moves out of superficial cortical layers, we intraperitoneally injected mice with mannitol before electroporation to decrease both the intracranial pressure and brain volume (given its diuretic effect) and to increase the extracellular space for spreading the DNA solution. Although the transfection efficiency improved slightly, the effect was limited to the visual cortex (3 positive sites/9 penetration sites).

### Distinct Projection Patterns of Thalamocortical Axons

**FIGURE 4:**
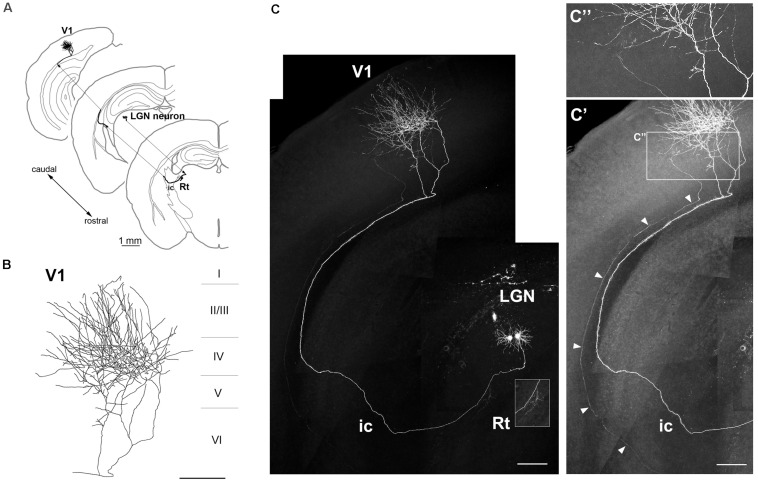
Axonal projection of an LGN neuron. **(A–C)** Axonal projection of an LGN neuron to V1 and thalamic Rt (inset in **C**). The neuron had a dense axonal arbor in layer IV **(B)**. Another dim ascending axon (arrowheads) was observed from the internal capsule (ic) to V1 (**C’**, brightness adjustment view of panel **C**), but in patches in layer IV of V1 (**C”**, higher magnification view of areas indicated in panel **C’**). Scale bars, 1 mm for panel **(A)**; 200 μm for panels **(B,C,C’)**; 100 μm for panel **(C”)**.

**FIGURE 5:**
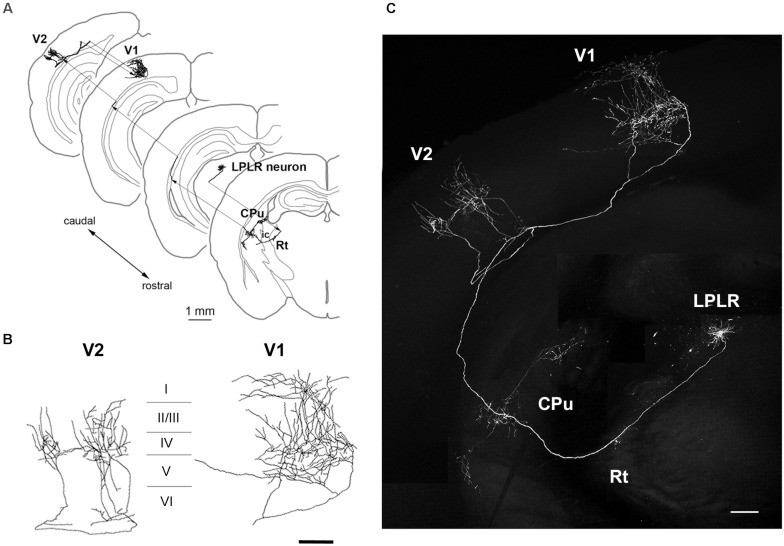
Axonal projection of an LP neuron. **(A–C)** Main targets of an LP neuron, including V1 and V2 as well as extravisual areas such as the caudate putamen (CPu). The LP neuron located in the laterorostral region of the LP (LPLR) had dense axonal arbor in layers I and V of V1 and layer IV of V2 **(B)**. Scale bars, 1 mm for panel **(A)**; 200 μm for panels **(B,C)**.

We examined whether axons extending from GFP-expressing neurons were clearly visible. By the third week after electroporation, thalamocortical axons were traceable from the cell body to the target area (Figures 4, 5). One GFP-expressing LGN neuron projected to the reticular nucleus (Rt) and layers IV and II/III of V1 along the visual pathway (Figures 4A–C). Although an adjacent GFP-positive neuron also projected its axon to V1 in parallel (Figure 4C), the fluorescence intensity was too weak for tracing and the axonal arbors were invisibly truncated in layer IV of V1, so that was distinguishable from the strongly expressing neuron (Figures 4C’,C”). In fact, only thalamic neurons strongly expressing GFP were observed to extend thalamocortical fibers and branches into cortical layers and/or subcortical regions (10 traceable neurons/14 projection neurons bearing GFP-positive axons extending through the internal capsule). Repeated (multisite) DNA injection (Figure 2A) slightly increased the fraction of traceable neurons (3/3 projection neurons) compared to single-site injection (7/11 projection neurons).

The LP harbors thalamocortical neurons that project to multiple regions in the cortex (Nakamura et al., 2015). Neuronal tracing revealed that one of the GFP-expressing LP neurons extended its axon to the Rt, caudate putamen, layer IV of the secondary visual cortex (V2), and layers I and V of V1 (Figures 5A–C), suggesting that it is a component of the extrageniculate pathway in the mouse visual system. Importantly, the LP included multiple neuronal subtypes with distinct patterns of projection to cortical layers and/or subcortical regions, including the striatum and amygdala (Clascá et al., 2012). Thus, single-cell labeling by electroporation enables visualization of long-range axonal projections and can identify different thalamocortical neuron subtypes.

### Localization of Presynaptic Proteins in Terminal Boutons

**FIGURE 6:**
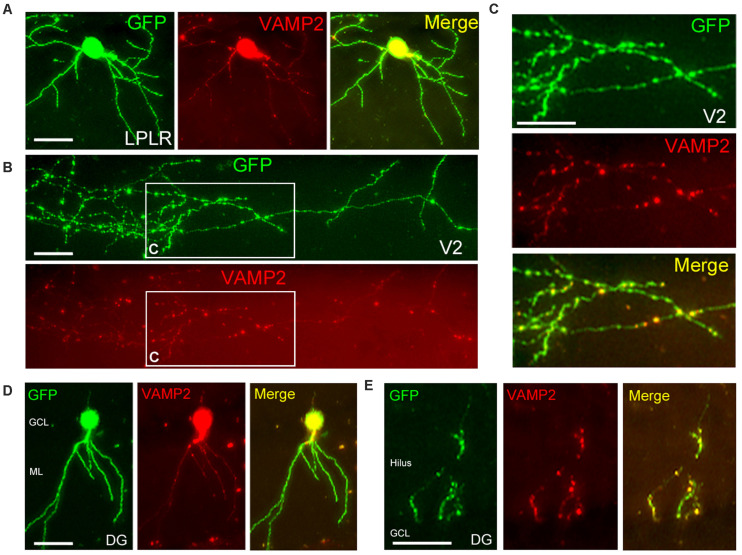
Localized distribution of presynaptic proteins *in vivo*. **(A–C)** Distribution of GFP (green) and VAMP2-mOrange (red) in a single neuron of the laterorostral region of the LP (LPLR). VAMP2 fusion protein was expressed at a low level in a few dendrites **(A)** but accumulated in the thalamocortical axon **(B)**, particularly in terminal boutons in layer I of V2 (**C**, higher magnification view of areas indicated in panel **B**). **(D,E)** Localization of GFP and VAMP2-mOrange within a DG granule cell. As in the LPLR neuron, VAMP2 fusion protein was densely distributed in the axon **(E)** but sparse in the dendrites **(D)**. GCL, granule cell layer; ML, molecular layer. Scale bars, 50 μm for panels **(A,D,E)**; 25 μm for panels **(B,C)**.

Time-lapse imaging of GFP-tagged presynaptic proteins has been carried out in cultured neurons, *Xenopus* retinal axons, or mouse corpus callosum axons *in vivo* (Ahmari et al., 2000; Hu et al., 2005; Ruthazer et al., 2006; Evans et al., 2019). To visualize thalamocortical axons and their presynaptic terminals, we co-electroporated two vectors encoding GFP and the axonal synaptic vesicle protein VAMP2 conjugated with mOrange into the thalamus or hippocampus. Similar to a previous study (Haas et al., 2001), both vectors were transfected into the same neuron in the postnatal mouse brain (Figures 6A–E; 7 co-expressing cells/7 transfected cells, all transfected cells expressed both GFP and VAMP2-mOrange). The expression of VAMP2-mOrange was strongly induced in the cell body of an LP neuron or granule cell along with GFP (Figures 6A,D); however, VAMP2-mOrange was less widely distributed in the dendritic arbor of these cells. The same LP neuron extended its axon to V2, in which we observed a number of terminal boutons strongly labeled with GFP (Figures 6B,C). Given their role in synaptic transmission, we examined VAMP2 distribution in the terminal boutons. As expected, VAMP2-mOrange was detected in the terminal boutons in layer I of V2 (Figures 6B,C) and the quantitative analysis revealed that VAMP2-mOrange was preferentially localized in axons than in dendrites (number of puncta per dendritic segment, 0.48 ± 0.06 vs. number of puncta per axon segment, 2.19 ± 0.30; 65–90 segments; *p* < 0.0001, Mann–Whitney *U* test). A similar VAMP2 distribution was observed in DG granule cells (Figure 6E; number of puncta per dendritic segment, 0.42 ± 0.10 vs. number of puncta per axon segment, 1.39 ± 0.18; 31–33 segments; *p* < 0.0001, Mann–Whitney *U* test). Thus, the presynaptic proteins introduced by our electroporation method were targeted to axons rather than to dendrites, possibly via the endogenous transport system (Cui-Wang et al., 2012).

## Discussion

In the present study we developed a single-cell electroporation method for labeling postmitotic neurons and their long-range axonal projections deep within the brain. We used this technique to simultaneously introduce two different genes of interest into the same neuron, demonstrating the utility of our technique for evaluating morphology and gene function in a single neuron in living animals.

Improvements in embryonic electroporation over the last two decades have allowed temporally and spatially restricted control of gene expression in cells–for example, pulse-labeling of a cell population depending on its date of birth (Sugiyama and Nakamura, 2003) and microelectroporation to restrict the area of transfection (Kataoka and Shimogori, 2008). Additionally, vectors encoding Tet on/off and Tol2 transposon components have enabled conditional expression of genome-integrated transgenes for gain- and loss-of-function studies (Sato et al., 2007; Hou et al., 2011). In ferret, *in utero* electroporation has been used in conjunction with the Cre/loxP and CRISPR/Cas9 systems for persistent gene activation or repression in the postnatal brain (Matsui et al., 2013; Shinmyo et al., 2017). In contrast, transfection of single cells remains technically challenging even in embryos.

Studies in embryos have revealed that the concentration of plasmid DNA around target cells is an important determinant of the transfection efficiency (Sugiyama et al., 2000). Our results confirmed that by increasing the amount of injected DNA, more neurons were labeled, and more clear morphology of single neurons was obtained, except in the cerebral cortex (Figures 2–5). Despite the cellular damage along the penetration tract, DNA injection with strong air pressure did not increase the number of damaged cells or decrease the survival rate of the transfected cells as compared to the results with weak pressure. Thus, for electroporation of postmitotic neurons, a higher vector concentration in the extracellular space increases the transfection efficiency.

Previous methods for single-cell electroporation have been based on the juxtacellular recording-labeling technique, using a high-resistance patch pipette (10–15 MΩ, 1–2 μm tip diameter) and weak air pressures (10–500 mbar) to approach in close proximity to the cell somata (for such pipette tip-cell contact, small electronic pulses, 1–2 μA are delivered) (Haas et al., 2001; Kitamura et al., 2008; Judkewitz et al., 2009; Oyama et al., 2013; Ohmura et al., 2015; Pagès et al., 2015). These techniques can combine single-cell labeling with recording, but an electrophysiological equipment and technical expertness are required, and the labeling efficiency is limited (approximately 30–40%) (Oyama et al., 2013; Ohmura et al., 2015). Visually guided single-cell electroporation with two-photon microscopy provides high success rates and cell specific labeling, however, it cannot be applied to neurons within deep brain regions (Kitamura et al., 2008; Judkewitz et al., 2009; Pagès et al., 2015). Here we present an approach to achieve high transfection efficiency using a sharp-shank pipette, in particular, to reach deep brain circuits. Compared to others, our approach is more easy and rapid (completed in 30–60 min), and it only requires simple equipment like a standard stereomicroscope and electroporator; a stimulus isolator can be substituted for an electroporator as described in previous studies (Uesaka et al., 2008; Judkewitz et al., 2009; Oyama et al., 2013; Ohmura et al., 2015). The embryonic system (CUY21 electroporator and electrodes) may be also applicable after injecting the DNA, considering the high transfection efficiency obtained with the ≥20 μm diameter pipette (Figures 2, 3). In contrast, our method has a limitation in cell specific transfection, and combining different systems (e.g., Cre/loxP systems) is required to restrict target cell types. Taken together, our approach is applicable to any regions and species amenable to injection of DNA solution.

Thalamic nuclei include a mixture of neuronal subtypes projecting to multiple target areas. At least 3 cell types (core-, matrix-, and intralaminar-type neurons) have been identified based on their axonal projections to cortical layers and/or subcortical structures (Jones, 2001; Clascá et al., 2012). According to this classification, the LGN cell in the present study is a core-type relay neuron extending to layer IV of V1 (Figure 4), while the LP cells are matrix-type neurons projecting to layer I of V1 or V2 (multilateral-and focal-type neurons in Figures 5, 6, respectively). LP nuclei containing core- and matrix-type neurons are reciprocally connected to both V1 and V2, in addition to receiving inputs from the superior colliculus and pretectal visual areas and sending outputs to the striatum and other cortical areas (Nakamura et al., 2015). Therefore, LP nuclei are regarded as higher-order relay thalamic nuclei (Sherman, 2016) that contribute to V2 function by transmitting converging information in the extrageniculate pathway (Andermann et al., 2011; Marshel et al., 2011; Tohmi et al., 2014; Bennett et al., 2019).

Despite the functional importance of thalamic nuclei, the diversity of thalamic neuron subtypes has not been well characterized in terms of development, gene expression, or functional properties (Clascá et al., 2012). Reasons for this include the following: (1) limited evidence garnered from single-axon reconstruction studies; (2) a lack of markers for different neuronal subtypes; and (3) the dispersion of neuronal subtypes across anatomically defined thalamic nuclei. Single-cell transcriptome analyses can potentially reveal the molecular profiles of recently reconstructed thalamic neurons (Winnubst et al., 2019), while our single-cell gene transfer technique can be used to analyze gene function in distinct neuronal subtypes to provide insight into their specific role(s) in neural circuits.

A major advantage of electroporation is that multiple genes (or shRNAs) can be simultaneously introduced into the same single neuron, permitting focal suppression or stimulation of neuronal activity *in vivo* by altering gene expression (e.g., potassium channel Kir2.1, light-gated algae channel channelrhodopsin; Judkewitz et al., 2009). As in imaging studies of GFP-tagged presynaptic proteins (Ahmari et al., 2000; Hu et al., 2005; Ruthazer et al., 2006; Evans et al., 2019), mOrange-tagged VAMP2 distributed in terminal boutons is useful for visualizing synaptogenesis according to neuronal activity in living animals.

Responses to sensory and converging stimuli vary across neuronal subtypes. As such, neural networks derived from a single neuron have been the focus of many studies: for example, in the cerebral cortex, retrograde tracing of a single neuron by electroporation of a recombinant rabies virus combined with 2-photon imaging allowed visualization of monosynaptic circuits (Wickersham et al., 2007; Kitamura et al., 2008; Marshel et al., 2010). Our single-cell electroporation technique provides an additional tool for observing monosynaptic projections, especially in deep brain areas. On the other hand, methods for anterograde transsynaptic tracing of a single cell require further development. The transsynaptic transport of wheat germ agglutinin (WGA)-lectin has been exploited to reconstruct cell populations-derived neural networks in transgenic mice (Yoshihara et al., 1999); anterograde tracing of a single neuron by electroporation of WGA-lectin construct could also be used in this manner. Additionally, single-cell transfection of genes encoding activity-dependent membrane-permeable molecules such as Otx2 and Arc (Sugiyama et al., 2008; Hou et al., 2017; Sakai et al., 2017; Pastuzyn et al., 2018; Erlendsson et al., 2020) is expected to reveal their mechanisms of transport between neuronal contacts. In conclusion, our method for manipulating single neurons in postnatal neural circuits provides a new approach for investigating cell-autonomous functions of genes and their association to neuronal morphology in the mammalian brain.

## Data Availability Statement

The datasets presented in this study can be found in online repositories. The names of the repository/repositories and accession number(s) can be found in the article/supplementary material.

## Ethics Statement

The animal study was reviewed and approved by the Committee for Animal Care at Niigata University.

## Author Contributions

SS and XH designed the expression vectors and *in vivo* experiments, and wrote the manuscript. JS, TI, XH, and SS performed the experiments and discussed the results of the experiments. JS, TI, and SS quantitated gene expression and performed histologic analysis. All authors contributed to the article and approved the submitted version.

## Conflict of Interest

The authors declare that the research was conducted in the absence of any commercial or financial relationships that could be construed as a potential conflict of interest.
